# Prognostic importance of prognostic nutritional index and modified Glasgow prognostic score in advanced lung cancer with targetable mutation

**DOI:** 10.1007/s00432-023-05529-w

**Published:** 2024-04-26

**Authors:** Burak Bilgin, Yunus Kuralay, Sebnem Yucel

**Affiliations:** 1 Department of Medical Oncology, Ankara Bilkent City Hospital, Ankara Yildirim Beyazit University, Cankaya, 06800 Ankara, Turkey; 2 Department of Internal Medicine, Erzurum Regional Education and Research Hospital, Erzurum, Turkey

**Keywords:** Nutrition, Inflammation, EGFR, ALK, Lung cancer

## Abstract

**Background:**

Inflammation and nutrition are important parameters that significantly affect survival in various malignancies. Prognostic nutritional index (PNI) and modified Glasgow prognostic score (mGPS) can reflect both inflammatory and nutritional conditions. Therefore, we aimed to evaluate the prognostic value of PNI and mGPS in patients who had the targetable mutation and also received targeted therapy.

**Materials and Methods:**

Advanced lung cancer patients with EGFR mutation (mut) and ALK rearrangement were enrolled to study, retrospectively. PNI has with the following formula: 10 × serum albumin (g/dl) + 0.005 × peripheral lymphocyte count (per mm^3^) and threshold value was accepted as 50. Modified GPS was also calculated using albumin and CRP level and patients were scored as range 0 to 2.

**Results:**

A total of 182 patients enrolled in the study. 132 and 50 of 182 patients had EGFR mut and ALK rearrangement, respectively. PFS was significantly longer in high PNI group in both the EGFR and ALK rearrangement-positive subgroups (*P* = 0.004 for EGFR mut-positive group; *P* = 0.017 for ALK rearrangement-positive group). Additionally, PFS was significantly shortened from mGPS 0 to 2 (*P* =  < 0.001 for EGFR mut-positive group; *P* = 0.016 for ALK rearrangement-positive group).

**Conclusion:**

Both PNI and mGPS can be used as a reliable, inexpensive, and easily applicable prognostic index in the advanced lung cancer patients who had the targetable mutation and also received targeted therapy.

## Introduction

Lung cancer is the most common type of cancer and also the leading reason for cancer-related death worldwide (Bray et al. 2018). Although there are many histological subgroups of non-small cell lung cancer (NSCLC), adenocarcinoma is the most common histological subtype. In the past years, our knowledge about lung cancer pathogenesis has been increased and many new mutations, which have an important role in tumor development also called as “driver mutation”, were discovered. Approximately, 25% of lung adenocarcinoma patients have targetable driver mutation including EGFR, ALK, ROS-1, BRAF, MET, and cErbB2. EGFR and ALK mutation can be seen between 12–17 and 5–7% of the lung adenocarcinoma, respectively (Zhu et al. 2017). Recently, many tyrosine kinase inhibitors (TKI) that targeted EGFR, ALK, ROS-1, BRAF, MET, and the others showed superior efficacy compared to chemotherapy in the previous trials. Therefore, the targeted therapies have been considered as the primary treatment options for advanced lung cancer with targetable mutation.

Prognostic nutritional index (PNI) which can be calculated easily using peripheral blood parameters is used for reflection of the nutritional and immunological condition. In previous studies, high pre-treatment PNI level was found as a good prognostic factor for various malignancies including gastric, colorectal, bladder, and also lung cancer (Migita et al. 2013; Jian-Hui et al. 2016; Peng et al. 2017; Li et al. 2018). Modified Glasgow prognostic score (mGPS) is another inflammation and nutritional-based prognostic scoring system. Modified GPS is calculated using serum C-reactive protein (CRP) and albumin level and ranges from 0 to 2. In previous studies, mGPS has been also detected as a prognostic marker in various malignancies including lung cancer (Jin et al. 2017; He et al. 2018; Jomrich et al. 2018). It is known that both nutritional and immunological status are closely related to long-term outcomes of lung cancer (Yu et al. 2017, Seo et al. 2019). Therefore, PNI and mGPS may be considered as the superior prognostic marker compared to the other peripheral blood-based index due to evaluation of both nutritional and inflammatory conditions.

Nowadays, the absence of generally accepted prognostic markers for targeted therapies is an important problem for the evaluation of the treatment course. Per our knowledge, there are very limited studies that investigated the prognostic value of PNI and mGPS in lung cancer with targetable mutation. Therefore, we aimed to investigate the prognostic effect of pre-treatment PNI and mGPS in advanced lung adenocarcinoma patients with targetable mutation and also treated with TKI.

## Materials and methods

All the patients diagnosed with advanced NSCLC were retrospectively reviewed in our hospital. Inclusion criteria were as follows: (1) ≥ 18 years old, (2) Stage 3C and 4 patients (according to 8th edition of American Joint Committee of Cancer Staging classification), (3) Have EGFR mutation (Exon 19 deletion and exon 21 L858R mutation) and also ALK-EML4 gene rearrangement, (4) Received erlotinib or gefitinib in EGFR mutation-positive patients and also crizotinib in ALK rearrangement-positive patients as first- or second-line treatment, (5) Available pre-treatment blood parameter data, (6) Adequate follow-up data. Exclusion criteria were also followed: (1) Have targetable mutation except EGFR and ALK, (2) Presence of active infection or autoimmune disease at the time of collection of the blood samples, (3) Previous or coexisting cancers other than NSCLC, (4) Received drugs that can be affect blood parameters, (5) have any protein-losing disease.

All data were collected retrospectively using hospital electronic database. PNI was calculated with the following formula to identified by Onodera and colleagues: 10 × serum albumin (g/dl) + 0.005 × peripheral lymphocyte count (per mm^3^) (Onodera et al. 1984). Modified GPS was also calculated using serum CRP and albumin level. Patients with both an elevated CRP (> 10 mg/l) and decreased albumin (< 3.5 mg/dl) are assigned a score of 2, whereas those with either an elevated CRP or decreased albumin alone are assigned a score of 1. Patients with a normal CRP and albumin level are assigned a score of 0. Blood samples were collected before the TKI initiation. ROC curve analysis was performed to determine the cut-off value for PNI and the ideal result was not obtained. For this reason, the median value was accepted as the cut-off. The threshold level of PNI was accepted as 50 and had both the median PNI levels of our population. Additionally, the population was stratified as low PNI (< 50) and high PNI (≥ 50) group. The patients were also stratified according to mGPS range 0–2.

The patient population was mainly separated two groups as EGFR mutation-positive and ALK rearrangement-positive. EGFR mutation-positive group was also stratified according to mutation subtypes (Exon 19 deletion and exon 21 L858R mutation). We evaluated the prognostic effect of PNI on both EGFR and ALK-positive subgroup, separately. The primary outcome of our study was progression-free survival. Tumor response was evaluated by CT scan or 18-FDG PET BT scan according to the Response Evaluate Criteria for Solids Tumors (RECIST) (Schwartz et al. 2016). Progression-free survival (PFS) was described as the time from initiation of crizotinib to RECIST-defined progression or death.

Categorical variables were compared using the chi-square or Fisher’s exact test. The variables were investigated using visual and Kolmogorov–Smirnov/Shapiro–Wilk test to determine whether or not they are normally distributed. Mann–Whitney U test and Spearmen test were used to compare non-normal or normally disturbed, ordinal variables, respectively. The difference in survival outcomes was investigated using the log-rank test. The Kaplan–Meier survival estimates were calculated. A *P*-value of less than 0.05 was considered to show a statistically significant result. Statistical analyses were performed using the SPSS software version 23.

## Results

### Study population

A total of 182 patients were included in this study. 132 and 50 of 182 patients were EGFR mutation-positive and ALK rearrangement-positive, respectively. In the EGFR-positive group, the rates of exon 19 deletion and exon 21 L58R mutation were 53% and 47%, respectively. In the EGFR-positive population, 62 of 132 (47%) were PNI ≥ 50, and 70 of 132 (53%) were also PNI < 50. In addition, 52 patients (39.2%) were mGPS 0, 63 patients (47.7%) were mGPS 1, and 17 patients (13.1%) were mGPS 2. In ALK-positive population, 21 of 50 (42%) patients were PNI ≥ 50 and 29 patients (58%) were PNI < 50. Among them, 20 patients (40%) were mGPS 0, 22 patients (44%) were mGPS 1, and 8 patients (16%) were mGPS 2.

The median age of EGFR and ALK-positive patients was 63 (min–max: 21–86) and 51.5 (min–max: 26–76), respectively. The rate of male and female patients in EGFR-positive group was 44.3% and 55.7% and also in ALK-positive group was 56% and 44%, respectively. Demographic and baseline clinical characteristics were balanced across in both PNI ≥ 50 and < 50 and also mGPS 0, 1, and 2 groups. There is no statistically significant difference between these groups. The detailed comparison of demographic and clinical data of EGFR and ALK-positive patients according to PNI and mGPS is shown in Table 1 and 2, respectively.

**Table 1:** Demographic and clinical features of EGFR mutant NSCLC patients according to PNI level and mGPS

Parameter	PNI			mGPS			
	< 50	≥ 50	*P*	0	1	2	*P*
Age (Median, min–max)	64 (21 – 84)	62 (32 – 86)	0.34	61(37–83)	63.5 (32–86)	67 (21–77)	0.78
Sex (M/F) (%)	43.9/56.1	43.1/56.9	0.92	35.3/64.7	44.3/55.7	70.6/29.7	0.40
EGFR Mut			0.275				0.535
Exon 19del	48.6	58.1		47.1	56.5	58.8	
Exon 21 L858R	51.4	41.9		52.9	43.5	41.2	
Smoking (%)			0.60				0.59
Active	10.6	8.5		7.8	8.1	17.6	
Former	24.2	32.2		25.5	30.6	35.5	
Non-smoker	65.2	59.3		66.7	61.3	47.1	
ECOG PS (%)			0.21				0.10
0	1.5	1.7		0	3.3	0	
1	46.2	66.1		58.8	55.7	58.8	
2	46.2	30.5		37.3	39.3	23.5	
3	6.1	1.7		3.9	1.6	17.6	
Brain Met (Y/N- %)	10.6/89.4	18.6/81.4	0.201	17.6/82.4	12.9/87.1	11.8/88.2	0.72
Liver Met (Y/N- %)	15.2/84.8	15.3/84.7	0.98	15.7/84.3	12.9/87.1	23.5/76.5	0.55
Adrenal Gland Met (Y/N- %)	27.3/72.7	15.3/84.7	0.103	21.6/78.4	21/79	29.4/70.6	0.75
Lung Met (Y/N- %)	39.4/60.6	54.2/45.8	0.097	47.1/52.9	45.2/54.8	41.2/58.8	0.91
Bone Met (Y/N- %)	54.5/45.5	40.7/59.3	0.121	43.1/56.9	41.9/58.1	58.8/41.2	0.44
Pleura Met (Y/N- %)	45.5/54.5	42.4/57.6	0.72	47.1/52.9	50/50	41.2/58.8	0.8

*M* male, *F* Female, *Met* metastasis, *ECOG PS* Eastern Cooperative Oncology Group Performance score, *Y* yes, *N* no, *mGPS* modified Glasgow prognostic score, *PNI* Prognostic nutritional index, *Mut* mutation, *mGPS* Modified Glasgow prognostic score

**Table 2:** Demographic and clinical features of ALK mutant NSCLC patients according to PNI level and mGPS

Parameters	PNI			mGPS			
	< 50	≥ 50	*P*	0	1	2	*P*
Age (Median, min–max)	52 (31–76)	49 (26 – 70)	0.387	52.5 (35–74)	52 (26–76)	40 (31–58)	0.087
Sex (M/F) (%)	65.5/35.5	42.9/57.1	0.11	45/55	68.2/31.8	50/50	0.29
Smoking (%)			0.43				0.49
Active	4.3	5.6		15	10.5	12.5	
Former	34.8	16.7		20	21.1	37.5	
Non-smoker	60.9	77.8		65	68.4	50	
ECOG PS (%)			0.23				0.68
0	7.8	6.1		2.2	4.5	1.4	
1	60.2	55.9		65.4	62.8	59.7	
2	30.9	35.6		29.8	30.5	36.4	
3	1.1	2.4		3.6	2.2	2.5	
Brain Met. (Y/N- %)	32/68	40/60	0.57	38.9/61.1	31.6/68.4	37.5/67.5	0.89
Liver Met. (Y/N- %)	17.9/82.1	9.5/90.5	0.409	10.5/89.5	18.2/81.8	12.5/87.5	0.77
Adrenal Gland Met. (Y/N- %)	55.2/44.8	28.6/71.4	0.061	40/60	50/50	37.5/62.5	0.74
Lung Met. (Y/N- %)	20.7/79.3	19/81	0.88	10/90	31.8/68.2	12.5/87.5	0.17
Pleural Met. (Y/N- %)	27.6/72.4	28.6/71.4	0.93	35/65	22.7/77.3	25/75	0.66
Bone Met. (Y/N- %)	37.9/62.1	33.3/66.7	0.73	35/65	27.3/72.7	62.5/37.5	0.202

*M* male, *F* Female, *Met* metastasis, *ECOG PS* Eastern Cooperative Oncology Group Performance score, *Y* yes, *N* no, *mGPS* modified Glasgow prognostic score, *PNI* Prognostic nutritional index

### Survival analysis according to PNI and mGPS

**Fig. 1:**
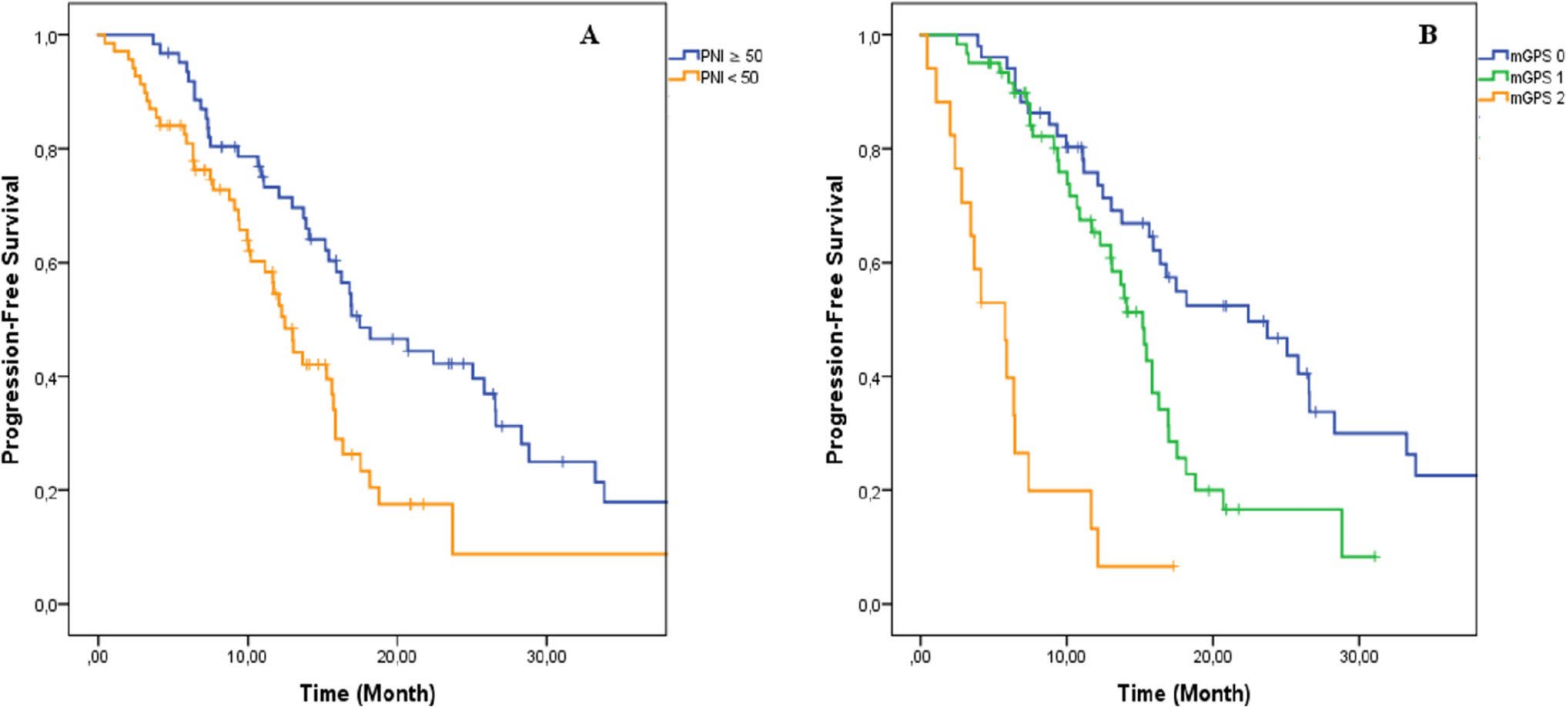
The progression-free survival graphics according to PNI and mGPS. **A** Prognostic nutritional index, **B** Modified Glaskow Prognostic Score

The median follow-up for PFS was 15.2 months for EGFR-positive patients and 15.1 months for ALK-positive patients. In EGFR-positive subgroup, PFS was significantly longer in PNI ≥ 50 than PNI < 50. In the patients with PNI ≥ 50 and < 50, the median PFS was 17.47 months and 12.45 months, respectively (Fig. 1). In PNI ≥ 50 group, the risk for disease progression or death was lower approximately 50% compared with PNI < 50 group (Hazard ratio [HR]: 0.51, 95% CI 0.33–0.80; *P* = 0.004). In EGFR exon 19 deletion positive subgroup, median PFS was nearly significant longer in PNI ≥ 50 than < 50 (median PFS: 16.9 months vs. 12.09 months, *P* = 0.098). The median PFS was also significantly longer in PNI ≥ 50 group in the patients with EGFR exon 21 L858R mutation (median PFS; 18.1 vs. 13.4, *P* = 0.007). When the survival was evaluated according to mGPS, PFS significantly shortened from mGPS 0 to 2. The median PFS was 22.4 months, 15.1 months, and 5.7 months in the patients with mGPS 0, 1, and 2, respectively (*P* =  < 0.001) (Fig. 1). The risk of disease progression or death was approximately 2.5 times higher in patients with mGPS 1 and 2 than mGPS 0 (HR: 2.43; 95% CI, 1.51–3.92; *P* =  < 0.001). In subgroup analyses, median PFS significantly shortened from 0 to 2 in EGFR exon 19del and exon 21 L858R mutation. In EGFR exon 19del subgroup, median PFS was 22.4 months, 13.8 months, and 3.6 months in mGPS 0, 1, and 2, respectively (*P* =  < 0.001). In EGFR exon 21 L858R subgroup, median PFS was 23.6 months, 15.1 months, and 5.7 months mGPS 0, 1, and 2, respectively (*P* =  < 0.001). Multivariate analysis was also performed and potential related parameters (age, sex, and ECOG performance status) with PNI and mGPS were included to the analyses (multivariable analysis included parameters that were significant or closest to significance in the univariate analysis). As a result, both PNI and mGPS were found as an independent prognostic factor (Table 3).

**Table 3:** Multivariate analyses of potential prognostic factor for EGFR mutant advanced NSCLC

Parameter	Hazard ratio	*P*	95% CI
PNI	1.56	0.05	1.01–2.51
mGPS	2.78	< 0.01	1.74–3.81
Age	0.97	0.07	0.95–1.002
Sex	1.2	0.62	0.96–1.43
ECOG PS	0.89	0.53	0.63–1.27

*PNI* Prognostic Nutritional Index, *mGPS* Modified Glasgow Prognostic Score, *ECOG PS* Eastern Cooperative Oncology Group Performance score, *NSCLC* non-small cell lung cancer

**Fig. 2:**
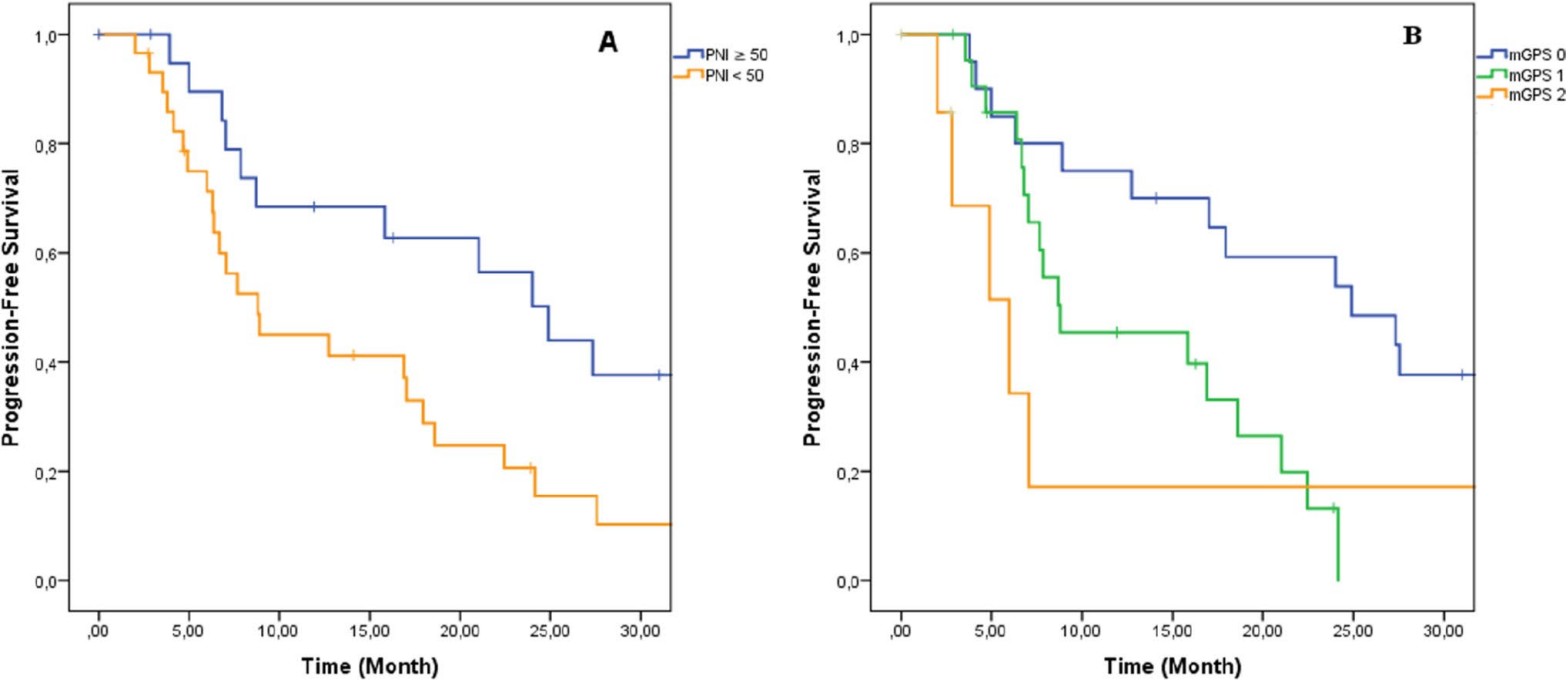
The overall survival graphics according to PNI and mGPS. **A** Prognostic nutritional index, **B** Modified Glaskow Prognostic Score

In ALK-positive patients, PFS was significantly longer in patients with PNI ≥ 50 than PNI < 50. The median PFS was 24.9 months in PNI ≥ 50 and 8.8 months in PNI < 50 (HR for regression or death: 0.42; 95% CI 0.21–0.86; *P* = 0.017) (Fig. 2). In addition to PNI, PFS was significantly shortened from mGPS 0 to 2. The median PFS was 24.9 months in mGPS 0, 8.8 months in mGPS 1, and 5.9 months in mGPS 2 arms, respectively (P = 0.016) (Fig. 2). The risk of disease progression or death was 2.7 times higher in GPS 0 arm than mGPS 1 and 2 arms (HR: 2.7; 95% CI 1.32–5.78; *P* = 0.007). The multivariate analysis was also performed, and both of PNI and mGPS were also found as an independent prognostic factor (Table 4).

**Table 4:** Multivariate analyses of potential prognostic factor for ALK mutant advanced NSCLC

Parameter	Hazard ratio	*P*	95% CI
PNI	1.76	0.043	1.13–2.98
mGPS	2.54	< 0.01	1.45–3.62
Age	0.88	0.1	0.81–1.12
Sex	1.1	0.65	0.91–1.23
ECOG PS	0.96	0.76	0.88–1.17

*PNI* Prognostic Nutritional Index, *mGPS* Modified Glasgow Prognostic Score, *ECOG PS* Eastern Cooperative Oncology Group Performance score, *NSCLC* non-small cell lung cancer

## Discussion

It is known that nutritional status and inflammation have an important role in treatment response and long-term outcome in various malignancy including lung cancer. In this study, we investigated the prognostic significance of PNI and mGPS, which are important parameters in the evaluation of inflammation and nutrition together, in patients with targetable mutations. In EGFR and ALK mutant patients, both PNI and mGPS were found to be a prognostic marker for PFS. High PNI and mGPS scores were associated with prolonged survival in both EGFR and ALK mutant patients. Similarly, both PNI and mGPS were found to be independent prognostic markers in multivariate analyses.

PNI has been used as a prognostic factor for various malignancy for a long time. PNI can reflect both nutritional and inflammatory conditions together rather than the other parameters which are reflected nutritional or inflammatory condition alone. Therefore, PNI has been considered as an important prognostic marker for malignant diseases. It can be calculated easily using simple, objective, and inexpensive laboratory parameters. In previous studies, PNI was found as a significant prognostic index for advanced or early-stage NSCLC. In these trials, high PNI level was found related to increased survival (Sheng et al. 2016; Xu et al. 2017, Seo et al. 2019). On the other hand, there is only one trial that investigated prognostic effect of PNI on advanced NSCLC with targetable mutation. Sheng et al. showed that high PNI was related to increased overall survival in patients with EGFR mutation. However, in subgroup analyses of this study, PNI was found as a significant prognostic marker for exon 21 L858R mutation but not exon 19 deletion. An important problem for investigation of PNI as a prognostic marker is the determination of the threshold level for PNI. The widespread consensus about the optimal threshold level of PNI has not been reached yet. In previous studies, PNI level for threshold varied from 45 to 52 (Qiu et al. 2015; Li et al. 2018; Wang et al. 2020). ROC curve analysis was performed to determine the cut-off value for PNI and the ideal result was not obtained. For this reason, we accepted that the threshold level was 50 as a median PNI level of our population and the patients were stratified as high (≥ 50) or low PNI (< 50).

In our trial, we found that high PNI level was related with significant longer PFS in the whole group and EGFR exon 21 L858R subgroup but not in EGFR exon 19del subgroup. These results were consistent with previous trial as mentioned above. The possible reasons for not reaching statistical significance in EGFR exon 19 del subgroup may be relatively low patient count or patients clinical features. On the other hand, we cannot explain the exact mechanism. To the best of our knowledge, this is the first study that investigates the prognostic value of PNI in both EGFR and ALK mutated patients treated with targeted agents. As mentioned above, both poor nutritional status and increased inflammation had a negative effect on survival due to various mechanisms. Our results found consistent with previous knowledge about the relationship between nutritional or inflammation and survival outcomes.

Modified GPS is another index that can reflect both inflammatory and nutritional conditions, and it can be calculated easily using serum albumin and CRP level. There are many studies and meta-analysis concerning mGPS and prognosis in various malignancies including gastric, colorectal, and also lung cancer without driver mutation (Woo et al. 2015; Zhang et al. 2015; Jin et al. 2017). All of these data showed that mGPS had a significant prognostic value in these malignancies. Additionally, survival has been significantly worsened from score 0 to 2. In a meta-analysis, the relative risk of death increased 1.77 times in lung cancer patients with high mGPS (Jin et al. 2017). To the best our knowledge, despite there are many studies that investigated mGPS in advanced lung cancer, there are no previous studies about advanced lung cancer that treated with targeted agents. Therefore, this is the first study that investigated prognostic value of mGPS in advanced lung cancer patients who had the targetable mutation and received targeted therapy. In our study, the results were consistent with previous trials that made in lung cancer without driver mutation. The survival worsened in patients with high mGPS in both EGFR and ALK-positive subgroup. Unlike PNI, the prognostic significance of mGPS showed in both EGFR exon 19 del and exon 21L858R subgroups. Additionally, the relative risks for progression or death were 2.5 and 2.7 times higher in mGPS ≥ 1 than 0 in EGFR and ALK mutated patients, respectively. As a result, mGPS can be accepted as a reliable, inexpensive, and easily applicable marker for EGFR and ALK mutated patients who were treated with targeted therapy.

The retrospective design of the study is the major limitation of our study and it may be related to selection bias of the patients. However, the number of patients may be sufficient for these rare subgroups of advanced lung cancer. Although the comorbidities that can directly affect albumin, CRP and CBC parameters are excluded in this study, and the unpredictable comorbidities can affect blood parameters indirectly. Another limitation of our study is the patients with the other targetable mutations, such as ROS-1, BRAF, and MET, not included in this study.

In conclusion, this is the first study that showed prognostic importance of PNI and mGPS as markers that reflect the inflammatory and nutritional conditions in advanced lung cancer with targetable mutation. Nowadays, the absence of reliable and easily applicable markers is an important problem for the management of patients with the targetable mutation. As a result of this study, PNI and mGPS as a reliable, inexpensive, and easily applicable prognostic marker can fill this gap. Additionally, these indexes may be guided to treatment selection in future. Therefore, further prospective studies are needed to demonstrate the possible effect of both PNI and mGPS on treatment selection.
